# Brazilian *Plasmodium falciparum *isolates: investigation of candidate polymorphisms for artemisinin resistance before introduction of artemisinin-based combination therapy

**DOI:** 10.1186/1475-2875-9-355

**Published:** 2010-12-08

**Authors:** Bianca E Gama, Natália K Almeida de Oliveira, José M de Souza, Fátima Santos, Leonardo JM de Carvalho, Yonne FC Melo, Philip J Rosenthal, Cláudio T Daniel-Ribeiro, Maria de Fátima Ferreira-da-Cruz

**Affiliations:** 1 Laboratory of Malaria Research, Instituto Oswaldo Cruz, Fiocruz, Rio de Janeiro (RJ), Brazil; 2 Ambulatory and Laboratory of Malaria Clinical Assays, Secretariat of Health Vigilance, Instituto Evandro Chagas, Belém (PA), Brazil; 3 Laboratory of Entomology, LACEN, Porto Velho (RO), Brazil; 4 La Jolla Bioengineering Institute, California, USA; 5 Fundação de Medicina Tropical do Amazonas, Manaus (AM), Brazil; 6 Department of Medicine, San Francisco General Hospital, University of California, San Francisco, USA

## Abstract

**Background:**

This study was performed to better understand the genetic diversity of known polymorphisms in *pfatpase6 *and *pfmdr1 *genes before the introduction of ACT in Brazil, in order to get a genotypic snapshot of *Plasmodium falciparum *parasites that may be used as baseline reference for future studies.

**Methods:**

Parasites from *P. falciparum *samples collected in 2002, 2004 and 2006-2007 were genotyped using PCR and DNA sequencing at codons 86, 130, 184, 1034, 1042, 1109 and 1246 for *pfmdr1 *gene, and 243, 263, 402, 431, 623, 630, 639, 683, 716, 776, 769 and 771 for *pfatpase6 *gene.

**Results:**

A *pfmdr1 *haplotype NEF/CDVY was found in 97% of the samples. In the case of *pfatpase6*, four haplotypes, wild-type (37%), 630 S (35%), 402 V (5%) and double-mutant 630 S + 402 V (23%), were detected.

**Conclusion:**

Although some polymorphism in *pfmdr1 *and *pfatpase6 *were verified, no reported haplotypes in both genes that may mediate altered response to ACT was detected before the introduction of this therapy in Brazil. Thus, the haplotypes herein described can be very useful as a baseline reference of *P. falciparum *populations without ACT drug pressure.

## Background

Resistance to anti-malarial drugs is one of the major obstacles to effective malaria treatment and control. Due to the worldwide spread of *Plasmodium falciparum *resistant to chloroquine and sulphadoxine-pyrimethamine, the World Health Organization (WHO) recommends the use of artemisinin-based combination therapy (ACT) as first-line malaria treatment. Artemisinin and its derivatives rapidly reduce clinical symptoms and parasite burden. Combination with a second agent improves treatment outcomes and minimizes the possibility of selecting artemisinin-resistant parasites [1].

ACT has demonstrated outstanding anti-malarial efficacy, but its long-term promise has recently come into question. First, *P. falciparum *field isolates from French Guyana and Senegal with markedly reduced *in vitro *sensitivity to artemether were reported [2], although other reports from a number of areas have not replicated these results [3-5]. Second, recent reports from Cambodia identified parasites with slightly reduced *in vitro *sensitivity, but also showed significant prolongation of parasite clearance times after treatment with artesunate [6,7]. These results lead to concern that parasites with diminished sensitivity to artemisinins may be spreading around the world.

The mechanism of decreased susceptibility of malaria parasites to artemisinins is uncertain. Gene amplification or specific point mutations in codons 86, 184, 1034, 1042 and 1246 of the *P. falciparum *multidrug resistance gene 1 (*pfmdr1*), have been associated with alterations in artemisinin sensitivity by genetic disruption, allelic replacement and *in vitro *experiments [8-11]. These polymorphisms also lead to diminished response to some other anti-malarials, including mefloquine and lumefantrine, and also enhanced response to chloroquine and amodiaquine [8,12,13]. There are also some indications for *pfmdr1 in vivo *allele selection [14-18], mainly N86, 184F and D1246, in reinfecting parasites after artemether-lumefantrine (AL) treatment, a fact that may constitute a first step toward resistance [19].

A second gene in which polymorphisms may mediate alterations in artemisinin sensitivity is that encoding a SERCA-type calcium-translocating ATPase known as *pfatpase6 *[2], the only ATPase identified in the *P. falciparum *genome. However, this gene is more diverse than previously thought [20] and evidence of the roles of *pfatpase6 *mutations, specially that at codon S769N, in decreased *in vitro *sensitivity to artemisinins is uncertain [21].

To better understand this diversity and to get a genotypic snapshot baseline for future studies, known polymorphisms in *pfatpase6 *and *pfmdr1 *genes were assessed in *P. falciparum *parasites before the introduction of ACT in Brazil. At that time, treatment regimen recommended to falciparum malaria by Malaria National Control Program (MNCP) was quinine plus doxycycline or mefloquine. From 2007, Brazilian MNCP has been implementing ACT (artemether-lumefantrine and artesunate-mefloquine) as first-line regimen for uncomplicated falciparum malaria.

In Brazil, genotyping of *pfmdr1 *gene is scarce and *pfatpase6 *analysis has just started. Two of such studies, conducted in 1998, evaluated polymorphisms on *pfmdr1 *gene [22,23] and the recent investigation of *pfatpase6 *gene mutations was carried out with *P. falciparum *parasites from a single Pará state locality where only 14% of the isolates studied were DNA sequenced [4].

## Methods

### Study sites, blood samples and DNA extraction

Parasites from 119 *P. falciparum *blood samples were evaluated. These samples were collected in different periods, at the time of diagnosis of uncomplicated falciparum malaria patients living in three Brazilian endemic areas: Porto Velho, Rondônia state (2002; n = 46); Paragominas, Pará state (2004; n = 19); and Manaus, Amazonas state (2006-2007; n = 54). Irrespective the locality, the casuistic comprised male young adults (mean age 30 years) with 1900 parasites/μL mean parasitaemia, that reported at least three previous malaria episodes. After obtaining informed consent, venous blood was collected according to protocols approved by the ethics research committees of Fiocruz, Fundação de Medicina Tropical do Amazonas, Instituto Evandro Chagas and Laboratory of Entomology. Pregnant women, indigenous people, prisoners and individuals less than 18 years of age were excluded. DNA was extracted from 1 ml of cryopreserved blood using QIAamp midi columns as described by the manufacturer (Qiagen).

### Characterization of polymorphisms

Relevant portions of the *pfmdr1 *and *pfatpase6 *genes were amplified using primers as previously described to amplify N86Y, E130K, Y184F, S1034C, N1042 D, V1109I and D1246Y *pfmdr1 *SNPs [24] and H243Y, L263E, L402V, E431K, A623E, A630 S, G639 D, N683K, K716R, K776N, S769N plus K771E *pfatpase6 *SNPs [25]. PCR products were separated by 2% agarose-gel electrophoresis. Products were purified through the Wizard SV Gel and PCR Clean-Up System (Promega), according to the manufacturer's instructions. Amplified fragments were directly sequenced using Big Dye^® ^Terminator Cycle Sequencing Ready Reaction version 3.1 (Applied Biosystems) on an ABI PRISM DNA Analyzer 3730 (Applied Biosystems) from the Genomic Platform/PDTIS/Fiocruz [26].

## Results

Only isolates that were successfully amplified at all regions of a gene were considered. In this light, *pfmdr1 *polymorphisms were evaluable in 85 samples (19 for Paragominas, 33 for Porto Velho and 33 for Manaus localities) and *pfatpase6 *polymorphisms in 65 samples (17 for Paragominas, 22 for Porto Velho and 26 for Manaus) for the most likely haplotype assembly. The amplification failures might have been due to primer sensitivity or to unexpected polymorphisms in target sequences, since all these samples generated DNA fragments using primers employed in malaria diagnosis based in conserved DNA regions [27]. Unsuccessfully PCR amplifications were already reported to *pfmdr1 *gene [28] and in relation to *pfatpase6 *gene at least 25% of the samples did not have evaluable SNPs [29].

Analysis of *pfmdr1 *gene revealed four single haplotypes. Three of these contained SNPs already associated with altered sensitivity to anti-malarial drugs (86Y, 184F, 1034C, 1042 D and 1246Y). One *pfmdr1 *haplotype NEF/CDVY (N86, E130, 184F, 1034C, 1042 D, V1109 and 1246Y) was present in 97% of samples. One parasite from Manaus displayed a wild type (3D7 strain) profile (Table 1). Concerning the *pfatpase6 *gene, four single haplotypes were found (HLLE/AAGNKKSK in 37% of samples, HLLE/ASGNKKSK in 35%, HLVE/AAGNKKSK in 5% and HLVE/ASGNKKSK in 23% of samples), displaying none, one (402 V or 630S) or two (402 V plus 630S) SNPs within the 12 positions analysed (H243Y, L263E, L402V, E431K, A623E, A630 S, G639 D, N683K, K716R, K776N, S769N and K771E). Mixed haplotypes were not seen in any samples.

**Table 1:** Pfmdr1 and pfatpase6 haplotypes from Paragominas (PRG), Porto Velho (PV) and Manaus (MAN) isolates.

Gene	Haplotypes	n (n/locality)	%	Mutated codons
*pfmdr1* (n = 85)	NE**F**/**CD**V**Y**	82 (19/PRG, 33/PV, 30/MAN)	97	4
	
	NE**F**/S**D**V**Y**	1 (MAN)	1	3
	
	**Y**EY/SNVD	1 (MAN)	1	1
	
	NEY/SNVD	1 (MAN)	1	0

*pfatpase6* (n = 65)	HLLE/AAGNKKSK	24 (6/PRG, 4/PV, 14/MAN)	37	0
	
	HLLE/A**S**GNKKSK	23 (11/PRG, 12/MAN)	35	1
	
	HL**V**E/AAGNKKSK	3 (PV)	5	1
	
	HL**V**E/A**S**GNKKSK	15 (PV)	23	2

Codon positions: *pfmdr1 *N86Y, E130K, Y184F, S1034C, N1042 D, V1109I, D1246Y (n = 85); *pftapase6 *H243Y, L263E, L402V, E431K, A623E, A630 S, G639 D, N683K, K716R, K776N, S769N, K771E (n = 65). The haplotype of 3D7 strain is underlined and the mutated codons are shown in bold characters.

A multilocus analysis was performed for 65 samples for which both *pfmdr1 *and *pfatpase6 *were evaluable. The associations found were (see Table 1 for description of haplotypes): NEF/CDVY + HLLE/ASGNKKSK (41%), NEF/CDVY + HLLE/AAGNKKSK (31%), NEF/CDVY + HLVE/ASGNKKSK (25%), NEF/CDVY + HLVE/AAGNKKSK (1.5%) and YEY/SNVD + HLLE/AAGNKKSK (1.5%).

Of the three studied localities, Paragominas and Porto Velho displayed only one *pfmdr1 *haplotype (NEF/CDVY), in contrast to Manaus, which presented some allelic variation (NEF/CDVY, NEY/SNVD, YEY/SNVD and NEF/SDVY). However, even in Manaus, NEF/CDVY was by far the most prevalent haplotype (91%). For *pfatpase6*, HLLE/ASGNKKSK was predominant in Paragominas (65%), HLVE/ASGNKKSK in Porto Velho (68%), and HLLE/AAGNKKSK and HLLE/ASGNKKSK haplotypes in Manaus (54% and 46%, respectively).

## Discussion

It was assessed mutations in two genes of *P. falciparum *parasites that have previously been implicated in mediating resistance to artemisinin and other anti-malarial drugs, from Rondônia, Pará and Amazonas states in Brazil. It was found a range of polymorphisms in both *pfmdr1 *and *pfatpase6*, although *pfmdr1 *gene was more polymorphic than *pfatpase6 *one.

Regarding *pfmdr1*, the majority of isolates from all sites displayed the NEF/CDVY allele that was first described in 1998 in *P. falciparum *isolates from Mato Grosso and Amapá states [22,23]. The NEF/CDVY haplotype might have been selected by extensive use of quinine in Brazil, as suggested by a reverse genetics experiment in which mutations 1034C + 1042 D + 1246Y were associated with decreased sensitivity to quinine [12]. In Manaus city, another haplotype, YEY/SNVD, was seen in one sample. This haplotype has previously been reported in Colombia and Guyana [30,31], but not in Brazil. The set of *pfmdr1 *haplotypes herein identified did not include the combination of N86, 184F and D1246 codons, already reported in patients with recurrent parasitaemia after AL treatment [14-18].

For *pfatpase6 *a few polymorphisms were detected as already observed in another Pará state locality [4], contrasting to those reported in Africa [15]. Importantly, the S769N mutation, which was previously reported to associate with decreased *in vitro *response to artemether in French Guiana, was not seen. In 35% of the isolates, only one SNP (630S) was detected in parasites from Paragominas and Manaus, and double-mutant parasites (402V + 630S) were noticed in 23% of the isolates, in parasites from Porto Velho. The 402V and 630 S mutations have also been detected in *P. falciparum *parasites from Africa [20,25,32] and elsewhere [33], but they were not observed in another study of Brazilian isolates from Tucuruí city, Pará state [4]. It is noteworthy that parasites from Paragominas, a rural and isolated locality, had the 630 S mutation, demonstrating that polymorphisms have a broad geographical range.

After an initial report linking a *pfatpase6 *polymorphism with altered response to artemether [2], the association between *pfatpase6 *SNPs and artemisinin response has been uncertain. Recent *in vivo *reports did not find associations between altered artemisinin responses and *pfatpase6 *polymorphisms [6,7,34]. Further, parasites selected for artemisinin tolerance did not contain *pfatpase6 *SNPs previously associated with altered artemisinin response [35]. Thus, the role of *pfatpase6 *polymorphisms is uncertain, but in any event a range of polymorphisms in both this gene and in *pfmdr1 *was observed in Brazil.

## Conclusions

In this study, although some polymorphism in *pfmdr1 *and *pfatpase6 *were verified, no reported haplotypes in both genes that may mediate altered response to ACT were detected before the introduction of this therapy in Brazil. Thus, the haplotypes herein described can be very useful as baseline reference of *P. falciparum *populations without ACT drug pressure.

## Competing interests

The authors declare that they have no competing interests.

## Authors' contributions

BEG participated in the design of the study, carried out the molecular analysis and drafted the manuscript; NKAO performed the PCR assays; JMS, FS and YFCM helped in design of the study and field facilities for blood sample collections; LJMC, PJR and CTDR helped in the design of the study and reviewed the manuscript; MFFC conceived the study, coordinated its design, and finalized the manuscript. All authors have read and approved the final text.
